# Potential Obstacle Detection Using RGB to Depth Image Encoder–Decoder Network: Application to Unmanned Aerial Vehicles

**DOI:** 10.3390/s22176703

**Published:** 2022-09-05

**Authors:** Tomasz Hachaj

**Affiliations:** Institute of Computer Science, Pedagogical University of Krakow, 2 Podchorazych Ave, 30-084 Krakow, Poland; tomekhachaj@o2.pl; Tel.: +48-126-627-845

**Keywords:** encoder–decoder network, depth prediction, RGB to depth mapping, obstacle detection, Unmanned Aerial Vehicles, deep neural network

## Abstract

In this work, a new method is proposed that allows the use of a single RGB camera for the real-time detection of objects that could be potential collision sources for Unmanned Aerial Vehicles. For this purpose, a new network with an encoder–decoder architecture has been developed, which allows rapid distance estimation from a single image by performing RGB to depth mapping. Based on a comparison with other existing RGB to depth mapping methods, the proposed network achieved a satisfactory trade-off between complexity and accuracy. With only 6.3 million parameters, it achieved efficiency close to models with more than five times the number of parameters. This allows the proposed network to operate in real time. A special algorithm makes use of the distance predictions made by the network, compensating for measurement inaccuracies. The entire solution has been implemented and tested in practice in an indoor environment using a micro-drone equipped with a front-facing RGB camera. All data and source codes and pretrained network weights are available to download. Thus, one can easily reproduce the results, and the resulting solution can be tested and quickly deployed in practice.

## 1. Introduction

Encoder–decoder (E-D) deep neural networks (DNN) are currently the primary tool in many digital image processing applications. Particularly popular in these applications are networks with U-Net architectures [1]. In U-Net networks, there are multiple connections of encoder layers with decoder layers through so-called skip connections. Skip connections are used to transfer image features from different levels of resolution of the encoder pyramid, increasing the approximation capability of the network. These types of architectures find application in the segmentation of plants [2], people’s clothes or hair [3], or, for example, medical images [4]. Another valuable property of E-D networks is the capacity for inter-modality mapping and prediction using images of one modality to estimate images of another modality. This applies, for example, to RGB image to eye-tracking-based saliency map prediction [5] or, for example, to RGB to depth image prediction.

In the past few years, there have been a number of interesting papers proposing E-D to predict the distances of objects in the image from the camera lens. The authors most often focus on two issues: increasing the accuracy of the prediction [6,7,8,9,10,11,12] and increasing the speed of the network [13,14,15,16]. Speeding up network performance is achieved by reducing the computational complexity, usually by reducing the number of parameters. Porting models to mobile devices requires the conversion of the weights to a smaller number of bytes (from 4 bytes to 2 or even 1 byte). Both reducing the number of model weights and reducing their size (bytes) often requires a trade-off with model accuracy. Methods that allow RGB-based depth prediction have many practical applications. They allow one to use a single RGB camera to estimate the distance to objects. This is important in situations when one wants to reduce the number of sensors and energy consumption and thus the cost of an embedded system. This finds its application, for example, in small Unmanned Aerial Vehicles (UAVs, drones). UAVs have shown great potential in many fields—for example, in agriculture [17] or various types of surveillance [18].

It is also possible to train depth estimation methods without using ground truth (depth) data. These methods are called “unsupervised” and can be treated as more generalized versions of depth estimation with depth measurements available for training. Several possible solutions to this problem have been proposed. There is a group of algorithms that use a video stream for this task by processing ego-motion [19,20,21]. On the other hand, unsupervised single-frame methods most often use images acquired from several cameras simultaneously and estimate disparity maps based on them [22,23,24,25]. For example, the network described in the paper [13] is an E-D similar to U-Net-like networks with multiple small decoders working at different resolutions, directly on a pyramid of images or features.

There have been many proposed approaches for obstacle detection and collision avoidance for UAVs that use mainly various object detection methods [26,27,28,29] or depth maps measured directly by depth/distance sensors [30,31] or stereo cameras [32]. The work of [33] uses a method that acquires a small set of images while a quadrotor is hovering, from which the authors compute a dense depth map. Due to the acquisition time, however, this method is not a real-time method. An extensive survey on obstacle detection and collision avoidance can be found in [34]. Obstacle detection is a different issue from the mono-ocular and multi-ocular SLAM [35]. SLAM is designed to localize and map the environment, and algorithms of this type eliminate, among other things, moving objects from the final map of the environment that could potentially be a source of collisions. Moreover, modern visual SLAMs use sparse feature maps [33,36], which do not map potential obstacles in the paths of vehicles.

UAVs are increasingly used in precision agriculture, both outdoors (in open fields) and indoors (in greenhouses). The materials presented in the study [37] highlight these applications and provide a comprehensive overview that emphasizes the importance of SLAM for UAV solutions in such applications.

Based on the literature, it can be noted that no work has been published that reports the use of RGB to depth mapping from an RGB camera as a data source for an algorithm that predicts potential UAV collisions in real time. In this work, an encoder–decoder network with real-time performance was proposed to estimate the distance map using only a single RGB image (single frame). This network was then used as part of an algorithm to detect objects that could be a potential source of collision for the UAV. Because of the fact that the proposed method uses a single RGB sensor, it can be used in low-cost, lightweight, and low-power-consumption UAVs equipped only with a video camera. It is not necessary to use LIDAR or any other type of distance sensor to detect a potential obstacle in the drone’s path. This is an important financial advantage over more expensive systems of more complex UAVs.

The entire solution was implemented and tested in practice in an indoor environment using a micro-drone equipped with a front-facing RGB camera. All data and source codes and pretrained network weights are available to download. Thus, one can easily reproduce the results, and the resulting solution can be tested and quickly implemented in practice.

## 2. Materials and Methods

In this section, the proposed DNN is introduced together with the training and validation datasets. Moreover, an algorithm for potential obstacle detection and its implementation with a UAV is described.

### 2.1. Depth Estimation Network

**Figure 1:**
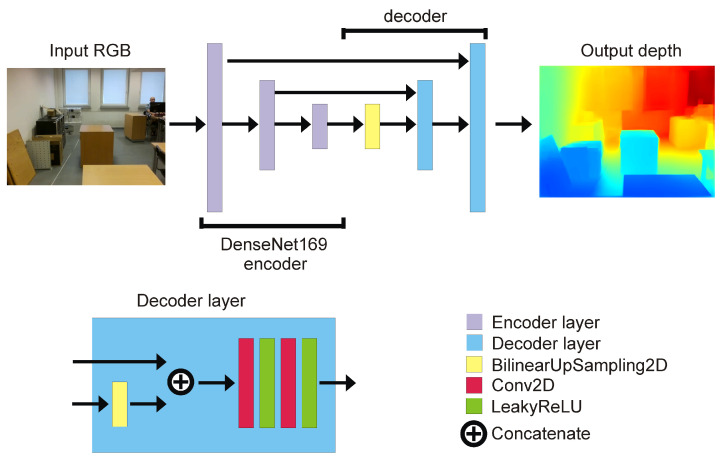
Deep encoder–decoder architecture of proposed network for RGB to depth image prediction.

The proposed network has been inspired by [6], which is a reliable and relatively fast architecture, albeit with too slow performance to be used in real-time solutions. In order to increase speed while preserving the efficiency of network performance, I propose an architecture that significantly reduces the number of DNN coefficients from 42.8 M (millions) to 6.3 M. The new proposed network is a U-net-inspired encoder–decoder with a DenseNet169 [38] backbone. DenseNet169 is pretrained on ImageNet [39]. Compared to the model proposed in the paper [6], the size of the E-D pyramid has been reduced from 4 to 2 skip connections. The output from the encoder is the fourth pooling layer of DenseNet169. The network architecture is presented in Figure 1.

Let us assume that $a_{i}$ is a ground truth depth image and ${\hat{a}}_{i}$ is an image with predicted depth values. Index i∈[1,..,n].

The utilized loss function, as in [6], is a three-element function using the following components:

- Point-wise depth loss for image index *i*:
  (1) $$l_{depth,i}=mean\left(\left|{\hat{a}}_{i}-a_{i}\right|\right),$$
  where mean(X) is the averaged value of matrix *X* elements.
- Edge-wise loss for image index *i*:
  (2) $$l_{edges,i}=mean\left(\left|\frac{\partial {\hat{a}}_{i}}{\partial x}-\frac{\partial a_{i}}{\partial x}\right|+\left|\frac{\partial {\hat{a}}_{i}}{\partial y}-\frac{\partial a_{i}}{\partial y}\right|\right),$$
- Structural similarity (SSIM) index [40] loss for image index *i*:
  (3) $$l_{ssim}=clip\left(\frac{1-ssim(\hat{a},a,maxdepth)}{2},0,1\right)$$
  where clip(x,0,1) is an element-wise value clipped to the range (0,1) and maxdepth is the maximal value of the depth pixel in the image.

The final form of the loss function is:

(4) $$l_{i}=w_{1}\cdot l_{ssim,i}+w_{2}\cdot \;l_{edges,i}+w_{3}\cdot l_{depth,i}$$

where $w_{1}=1$, $w_{2}=1$, $w_{3}=0.1$, as was recommended in [6].

Image augmentation during the training consists of color modification and mirroring.

### 2.2. Obstacle Detection

**Figure 2:**
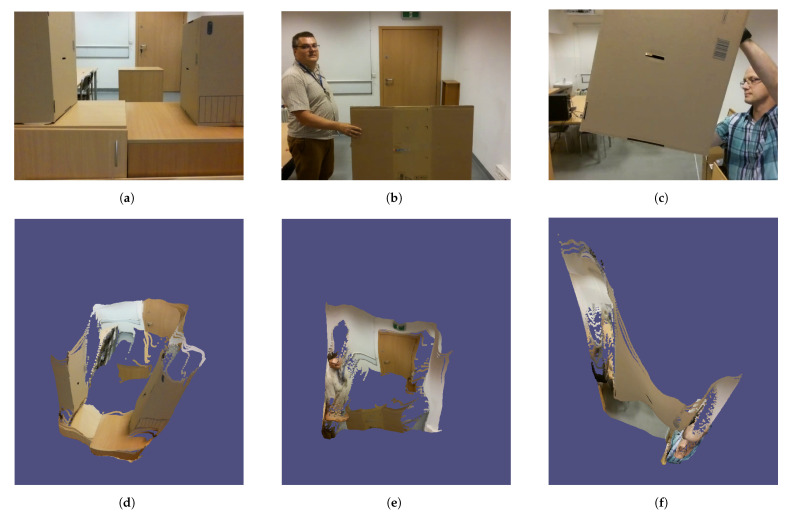
Examples of distance estimation inaccuracies visualized with point clouds. Top row (**a**–**c**) contains RGB image; bottom row contains depth estimation (**d**–**f**).

RGB to depth mapping networks are unfortunately not very accurate. This is perfectly evident in Figure 2, in which point clouds generated using the network proposed in Section 2.1 are presented. Unfortunately, the lack of stability of the image and the disturbances resulting from the incorrect estimation of the distance do not allow (yet) the use of any of the architectures of this type of network for direct and reliable distance measurement. Instead, networks of this type can estimate the mutual positions and distances of objects relative to the camera. Thanks to this, one can estimate which objects are farther away and which are closer. Based on this property, I propose a new algorithm to detect potential obstacles that are in front of the UAV. It consists of several steps, which include distance estimation using DNN, depth map clustering using the DBSCAN algorithm [41], and then using a series of thresholding with adaptive parameters to extract the object that is in a collision path with the drone, if such an object is present.

The algorithm proposed in this work, Algorithm 1, has the following parameters:

- rgb—image;
- scale—scaling parameter to lower computational complexity;
- (ϵ,μ)—DBSCAN parameters (epsilon and minimal number of samples);
- α—fraction of clusters to be considered foreground, where 1 means that all clusters are considered to be potential obstacles; 0.5 means that 50% of all clusters are taken as potential obstacles; clusters are ordered from the nearest to the camera to the farthest;
- β—maximal averaged distance of cluster elements from the camera, under which the cluster is considered as the foreground (potential obstacle), in range [0, 256], where 256 means that regardless of the averaged cluster distance from the camera, its elements are considered as foreground;
- ROI—region of interest in image that represents the further position of the UAV on its forward trajectory;
- η—if the fraction of non-zero values in ROI in the potential obstacle detection array is higher than η threshold, there is an obstacle on the UAV trajectory;
- dnn—deep neural network for depth image estimation from RGB image.

**Figure 3:**
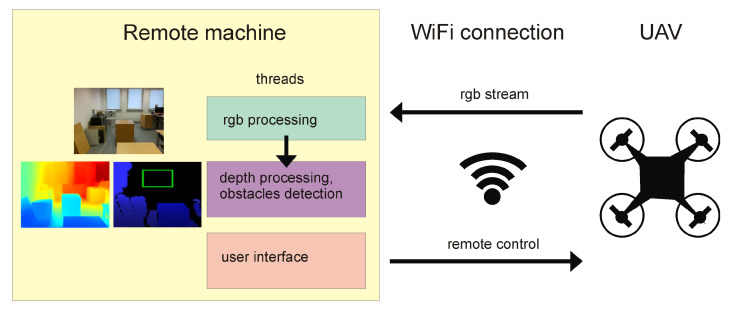
Diagram of the architecture of the system combining Algorithm 1 with UAV.

After testing, the following values were adopted to tune the parameters of the algorithm, which I will use for the rest of the work: scale=4, ϵ=5, μ=10, α=0.5, β=96, η=0.3. For an input image with a resolution of 640 × 480, the network returns a depth map with a resolution of 320 × 240. For scale=4, the DBSCAN segmented image has a resolution of 80 × 60. For this resolution, a 20 × 20 ROI was adopted, with its center point 20 pixels up from the center of the image. The ROI can be seen as a rectangle in Figures 3, 5 and 6. The rectangle is green if no obstacles are detected or red if obstacles are detected. Note that the horizontal stretch of the rectangle is an effect of visualization, in order to ensure that the image shape matches the figures.

### 2.3. Integration with Unmanned Aerial Vehicle System

For the real-time algorithm testing on the UAV, I have used a Tello drone, which is a popular platform to evaluate deep learning on UAVs due to the convenient API [42,43,44,45]. Tello enables real-time video streaming and remote control using the UDP protocol. The drone camera has been calibrated using a pinhole camera model using chessboard calibration patterns [46]. The UAV was an 80 g quadcopter with 3-inch propellers and a 1.1 Ah/3.8 V battery that supplies maximally 13 min of flight. The UAV is controlled over 2.4 GHz 802.11n Wi-Fi using the UDP protocol. The maximal speed is 8 m/s; however, because of inertia between steering commands and drone reaction caused by the communication protocol, I used 50% of the available speed to achieve a reliable reaction. The drone is equipped with a stationary 5 MP camera positioned in the front of the vehicle, which has an 82.6° field of view. The re-projection error, calculated as the absolute norm between undistortion transformation and the corner finding algorithm [46], equals 0.094.

The architecture of the system combining Algorithm 1 with the drone is shown in Figure 3. It consists of a drone communicating using WiFi with a remote machine that runs three threads: RGB processing thread, depth processing with obstacle detection thread (implementation of Algorithm 1), and user interface, which enables drone remote navigation for testing purposes. Source codes for this system are also available to download, together with all other algorithms introduced in this paper.
**Algorithm 1:** Algorithm of potential obstacle detection from RGB image
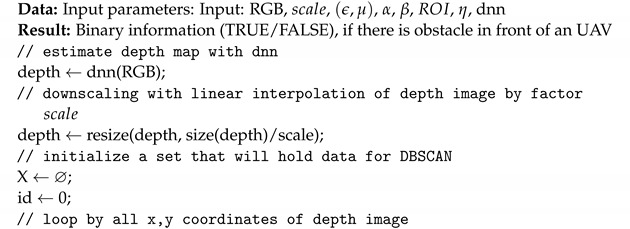

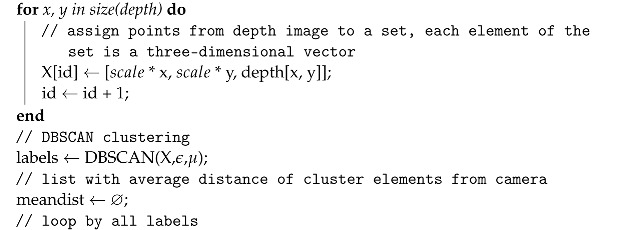

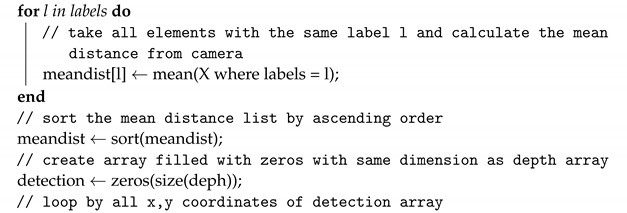

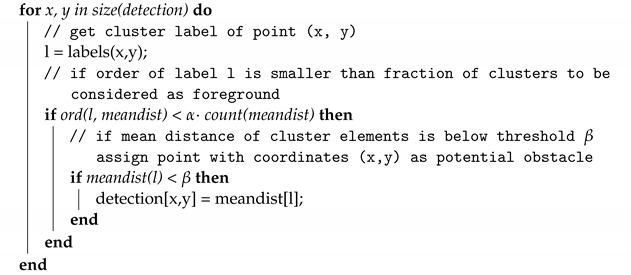

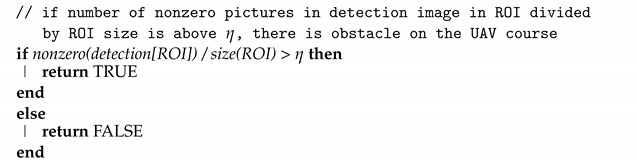


### 2.4. Dataset

The algorithm was tested in an indoor environment. This is because the drone that was used is not suitable for flying outdoors due to its small size. For this reason, the training set that I used consisted of pairs of RGB images and distance maps taken indoors. I used the NYU-Depth V2 dataset [47], which is composed of video sequences from a variety of indoor scenes as recorded by both the RGB and depth cameras. The dataset is available at: https://cs.nyu.edu/~silberman/datasets/nyu_depth_v2.html, accessed on 12 July 2022. The set has 50,688 training images and 654 test images.

## 3. Results

The network described in Section 2.1 has been implemented in Python 3.8. Among the most important libraries, Keras 2.8 with Tensorflow 2.8 for deep neural network modelling and calculation, and opencv-python 4.5.5 for general-purpose image processing have been used. I have utilized scikit-learn 1.0.2 for DBSCAN implementation. A pretrained model of VGG16 has been downloaded with the Keras Applications 1.0.8 package. All libraries were installed by PIP. Network training and evaluation was carried out on a PC equipped with an Intel i7-9700 3 GHz, 64 GB RAM, and an NVIDIA GeForce RTX 2060 GPU on Windows 10 OS. The CUDA support for Tensorflow was enabled. For the optimization of network weights, the stochastic gradient descent Adam optimizer [48] was utilized. The learning rate was set to $10^{-4}$ with batch size 2. The implementation is partially based on the source codes of paper [6], available at https://github.com/ialhashim/DenseDepth, accessed on 12 July 2022.

I have used third-party communication libraries to establish connection and video data streams from the drone using the DJITelloPy package https://github.com/damiafuentes/DJITelloPy, accessed on 12 July 2022. The proposed algorithm implementation, including the training algorithms, the evaluation algorithm, the drone application used during validation, and the application to generate video based on the results, can be downloaded from https://github.com/browarsoftware/tello_obstacles, accessed on 12 July 2022.

**Figure 4:**
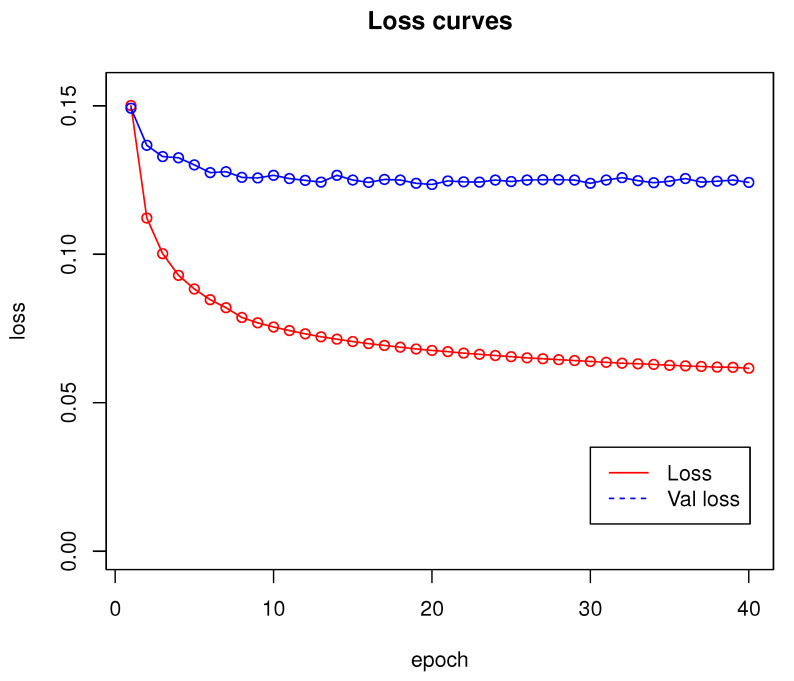
Loss curves for network introduced in Section 2.1. Training has been done using dataset from Section 2.4.

Network training was performed for 40 epochs and lasted approximately 53 h. Loss and validation loss results are shown in Figure 4.

The performance of the proposed network compared to other architectures that allow for distance (depth) map prediction is shown in Table 1. The metrics used were six metrics that are widely accepted to compare new methods against the state-of-the-art [49]:

- average relative error (lower is better):
  (5) $$REL=\frac{1}{n}\cdot \sum_{i}^{n}\frac{\left|\hat{a_{i}}-a_{i}\right|}{a_{i}},$$
- root mean squared error (lower is better):
  (6) $$RMS=\sqrt{\frac{1}{n}\sum_{i}^{n}{\left(\hat{a_{i}}-a_{i}\right)}^{2}},$$
- average ($log_{10}$) error (lower is better):
  (7) $$log_{10}=\frac{1}{n}\cdot \sum_{i}^{n}\left|log_{10}\left(\hat{a}\right)-log_{10}\left(a\right)\right|,$$
- threshold accuracy (higher is better):
  (8) $$\delta_{j}=\frac{\#(max\left(\frac{\hat{a}}{a},\frac{a}{\hat{a}}\right)<t_{j})}{n},$$
  where $t_{1}=1.25$, $t_{2}=1.25^{2}$, $t_{3}=1.25^{3}$.

The relatively small number of parameters of the network proposed in this work allowed it to significantly speed up its performance compared to the [6] architecture. The average processing time of a 640 × 480 resolution image for [6] is 0.104 (9.6 FPS) seconds and that of the proposed architecture is 0.058 seconds (17.2 FPS), on the hardware architecture whose specifications were given at the beginning of this section. This means that the proposed network has 6.79 times fewer parameters and runs 1.79 times faster. This performance is fast enough to achieve successful and reliable work with a 30 FPS video stream for the tested UAV. According to Table 1, the proposed network tends to have lower efficiency than architectures with more parameters, with the exception of [11], while it has the highest $\delta_{1}$ among architectures with a small number of parameters. Thus, the proposed architecture has a good trade-off between the number of parameters and the efficiency of operation. As can be seen, it is perfectly suitable for real-time system needs.

Based on the observations made during the experiments, the network is able to judge the distances of objects located at a minimum distance of approximately 20 cm from the camera. If the objects are closer, the network does not work properly recognize the objects’ textures as separate objects.

**Figure 5:**
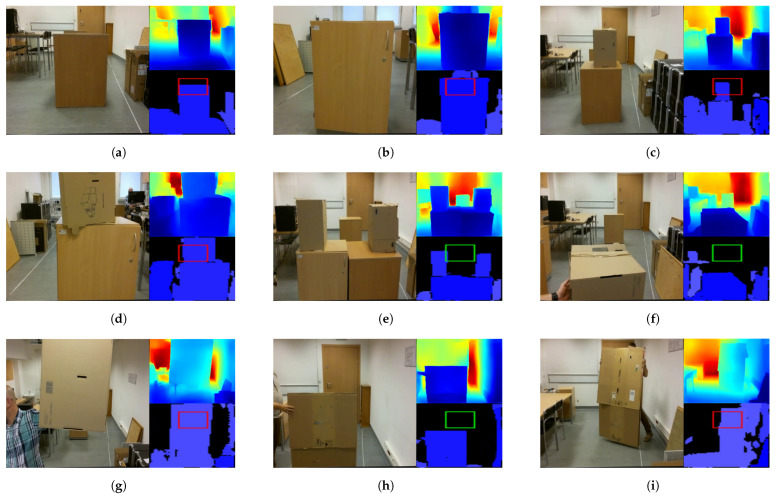
Different types of obstacles used during Algorithm 1 testing. In each sub-image, on the left is an RGB image, at the top right is a depth map estimated by proposed E-D, and at the bottom right are potential obstacles in the drone’s path as detected by Algorithm 1. If the rectangle is red, the algorithm predicts that the drone may collide with the obstacle. If the rectangle is green, the algorithm decides that there are no obstacles in the path. (**a**) Static obstacle 60 × 50 × 75 cm. (**b**) Static obstacle 60 × 40 × 80 cm. (**c**) Static obstacle with height 120 cm. (**d**) A second static obstacle with height 120 cm. (**e**) Static obstacle with a 45 cm deep “valley”. (**f**) Dynamic obstacle that is not on the drone’s flight trajectory. (**g**) A dynamic obstacle on a drone’s flight trajectory. (**h**) Dynamic obstacle that is not on the drone’s flight trajectory. (**i**) Dynamic obstacle on the drone’s flight trajectory.

In order to evaluate the potential obstacle detection algorithm, a set of 112 manually controlled test flights were performed, during which the drone encountered obstacles of various types. The on-board camera images during these flights were analyzed with the proposed Algorithm 1. The algorithm checked whether there were obstacles on the drone’s flight trajectory that threatened to collide with the drone if the drone continued its flight in that direction. Performance was evaluated using an approach similar to a confusion matrix. There were four possible situations: true positive (TP) means correctly detecting an obstacle that the drone could collide with if it continues to fly straight ahead. False positive (FP) means false detection of an obstacle that the drone could collide with if it continues to fly straight ahead. TP means that the drone could fly safely. True negative (TN) means no obstacle detection when there is no obstacle in the drone’s path. False negative (FN) means no detection of an obstacle in the drone’s path. During the experiment, the drone moved through an indoor space (laboratory room) that was 7.20 m long, around 2 m wide, and around 4 m high. The room was artificially lit, and the windows were covered with blinds. The room contained office furniture such as desks, boxes, chairs, etc. For safety reasons, the drone never moved towards an unshielded person. There were the following obstacles types in the drone’s path:

- Static obstacles of different sizes; see Figure 5a–e. In total, 34 flights were conducted, during which there were obstacles in the drone’s path as well as safe routes.
- Moving (dynamic) obstacles that appeared in front of the drone when it was not moving; see Figure 5f,g. Fifty-four tests on this type of obstacle were recorded. In 22 recordings, the moving obstacle did not cause a collision with the drone. In 32 recordings, the moving obstacle was on a collision track with the drone.
- Moving (dynamic) obstacles that appear in front of the drone—see Figure 5h,i—when the drone is moving forward. Twenty-four tests with this type of obstacle were recorded. In 12 of the recordings, the moving obstacle was in the drone’s path; in the other 12, the drone flew over the obstacle.

**Figure 6:**
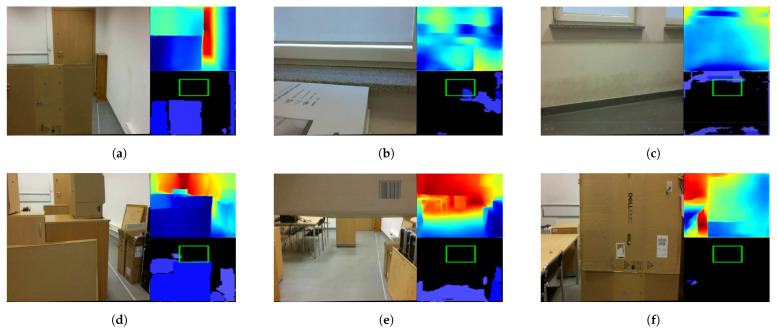
Example errors of Algorithm 1. (**a**) Misestimation of obstacle height. (**b**) Potential collision with a window. (**c**) Potential collision with a wall. (**d**) Misjudging the size of a complex obstacle. (**e**) Misjudging the distance to a dynamic obstacle from a hovering drone. (**f**) Misjudging the distance to a dynamic obstacle from a moving drone.

The drone was piloted toward these obstacles on both a collision course and a course to avoid them at safe altitudes. The drone also moved towards the walls, doors, and windows of the room. Walls, windows, and doors were also treated as obstacles that the algorithm should warn against. If Algorithm 1 correctly detected all obstacles, the flight was TP. If Algorithm 1 did not detect on the safe path of an obstacle, the flight was TN. If the algorithm made a mistake, it was FP or FN, respectively. A single mistake determined the rating of the algorithm in the corresponding flight as wrong (F). This means that, for each flight, the proposed algorithm receives one rating, correct (P) or wrong (F), indicating whether the algorithm detected all potential obstacles during it, as well as whether it made an FP error. Both erroneous and correct defections lasting less than 0.5 s were disregarded. This assumption was made because of observable artefacts in the camera images resulting from image noise. Evaluation of the results was done manually. All visualizations of the algorithm’s performance in the form of video recordings are available at https://github.com/browarsoftware/tello_obstacles, accessed on 12 July 2022. Sub-images in Figure 5 and Figure 6 are single frames of animation from these recordings. Drone camera data in the form of *.png files are available by contacting the author.

Overall, 16 of the 112 tests showed an Algorithm 1 error. One error was of type FP, when the height of a dynamic obstacle was incorrectly estimated; see Figure 6a. The remaining 15 errors were of type FN. Four times, the algorithm failed to detect a potential collision with a window—see Figure 6b—and once with a wall—see Figure 6c—and, once, it misjudged the structure of a two-part complex obstacle—see Figure 6d. The algorithm failed seven times to identify the appearance in front of a hovering drone of a dynamic obstacle with which it could potentially collide; see Figure 6e. Twice, it failed to identify the dynamic obstacle during flight with which it could potentially collide; see Figure 6f.

## 4. Discussion

According to the results presented in Section 3, Algorithm 1 proved to be an effective solution for the real-time detection of both stationary and moving obstacles. The loss function graph shown in Figure 4 shows that the training of the network proposed in Section 2.1 runs stably. The algorithm makes virtually no FP errors, as an error of this type appeared in <1% of all tested cases. Most of the errors made by the proposed algorithm were due to misjudgement of distance by the neural network. Note that the training set did not contain objects that would be placed in the air, as in Figure 6e, so the network may not have learned to recognize them correctly. Moreover, when the camera was too close to an object, the algorithm did not work properly (see Figure 6f). Such errors can be difficult to eliminate, because single-frame mono-ocular depth estimation cannot judge the scale of objects and can, with proper camera positioning, interpret, for example, a set of boxes lying on a table as a furnished room. Based on the observations made during the experiment, the minimum effective operating distance of the network is approximately 20 cm from an object. At smaller distances, the network gives erroneous results by not recognizing, for example, the surfaces of walls or doors. The training set also does not include detailed images of windows and window sills. For indoor solutions, they should be included in the training dataset.

The most important limitation of Algorithm 1 is that it is based on a network that does not accurately count distances, but only estimates them, allowing at most the determination of which objects are closer and which are further away. It is therefore necessary to use heuristics that include the parameters of the DBSCAN algorithm and the values of α, β, and η, which may vary slightly for different distance estimation networks and camera focal lengths. The proposed distance estimation algorithm was also tested on a Logitech HD 1080p webcam, and for identical algorithm parameters, the results for the distance estimation and segmentation of nearby objects were visually almost identical to those of the drone camera. Thus, it can be assumed that, for a network with sufficient performance as measured by (5)–(8), the selection of a depth-estimating E-D is not critical to the performance of the algorithm. By “sufficient”, I mean with measures (5)–(8) not worse than the network proposed in this paper. It is also possible that, if there are many objects at a short distance from an obstacle, e.g., <1.5 m, the DBSCAN algorithm will segment these objects and the threshold alpha will skip obstacles that are at a greater distance than 1.5 m in the drone’s path. However, this case did not occur in practice in the experiment. As was mentioned, the most important limitation of Algorithm 1 is that it is based on a network that does not accurately count distances, but only estimates them. Unfortunately, not only Algorithm 1 but all up-to-date E-D networks for single-frame depth estimation cannot be used as reliable sources of distance measurements for SLAM algorithms. All single-frame E-D networks generate similar inaccuracies to those that are presented in Figure 2.

The UAV on which Algorithm 1 was tested is designed to fly in indoor environments; however, the room in which I performed the tests was large enough that its walls were not a significant obstacle. The usefulness of Algorithm 1 in outdoor environments should be tested on another UAV; however, in my opinion, there is no contraindication for Algorithm 1 to be used also in outdoor environments.

## 5. Conclusions

The algorithm for potential obstacle detection using only an RGB camera applied to Unmanned Aerial Vehicles presented in this work has proven to be an effective and efficient method. To the best of my knowledge, this is the first proposed use of a deep encoder–decoder neural network in an application that allows small drones equipped only with an RGB camera to estimate a dense distance map and detect potential obstacles in real time. The algorithm evaluation results presented in Section 3 and Section 4 prove that the proposed method is reliable in the task of detecting potential obstacles. With open-source code available for download and a ready-made Python project structure, the method can be the first choice for both researchers and industry. All these facts might make the proposed approach the preferred algorithm for use in small, low-power-consuming UAV systems with a limited number of sensors. There are several subjects worth further research. First, it would be beneficial to optimize the algorithm for energy savings so that it can be used in an embedded UAV system. In this case, it may require the use of a slightly larger drone than the one used in the experiment described in this work. The second favorable eventuality is to transfer the computation to a portable system based on microcomputers, which are now perfectly capable of handling complex image processing [50]. For E-R RGB to depth mapping networks, a TPU co-processor such as Edge [51] may be required to achieve the appropriate operating frequency. The bottleneck is DBSCAN, which executes relatively slowly relative to the other elements of Algorithm 1. It would be necessary to test a number of available modifications of this algorithm for their effectiveness [52,53,54] as an alternative to the “classic” DBSCAN.

## Figures and Tables

**Table 1:** Comparison of performance of various depth estimation neural networks on the NYU-Depth-v2 dataset. The results are reported from the original papers. Last row is the results of the proposed network. The second column shows numbers of parameters in millions (M).

Method	#Params (M)	$\delta_{1}↑$	$\delta_{2}↑$	$\delta_{3}↑$	REL ↓	RMS ↓	${log}_{10}↓$
Eigen D. et al. [11]	-	0.769	0.950	0.988	0.158	0.641	-
AdaBins [9]	78	0.903	0.984	0.997	0.103	0.364	0.044
Alhashim I. and Wonka P [6]	42.8	0.846	0.974	0.994	0.123	0.465	0.053
Fu H. [12]	-	0.828	0.965	0.992	0.115	0.509	0.051
Huang K. et al. [8]	32.4	0.859	0.972	0.993	0.122	0.459	0.051
X. Tu et al. [16]	5.0	0.782	-	-	-	0.572	-
Wofk D. et al. [15]	3.9	0.775	0.911	0.972	0.211	0.599	0.079
Kerim Yucel M. et al. [14]	2.6	0.790	-	-	-	0.564	-
This method	6.3	0.819	0.965	0.992	0.139	0.587	0.059

## Data Availability

Source codes can be downloaded from: https://github.com/browarsoftware/tello_obstacles accessed on 12 July 2012.
